# Retrospective, single-center analysis of autoimmune hepatitis in Jordanian children: clinical features, treatments, and outcomes

**DOI:** 10.1186/s12887-024-04590-9

**Published:** 2024-02-08

**Authors:** Eyad Altamimi, Dana Al Omari, Hanadi Obeidat, Kamleh Barham

**Affiliations:** Pediatric and Neonatology Department, Faculty of Medicine, Jordan University of Science and Technology, Irbid, Jordan

**Keywords:** Liver, Children, Hepatitis, Immune, ANA, AMA, Liver failure

## Abstract

**Objectives:**

This study describes clinical, biochemical, and histological features and long-term outcomes in pediatric patients diagnosed with autoimmune hepatitis (AIH) at King Abdullah University Hospital, Jordan.

**Design:**

Retrospective, single-center study.

**Setting:**

King Abdullah University Hospital, Jordan.

**Participants:**

Inclusion of all pediatric patients with AIH diagnosed at our hospital from 2015 to 2023. Exclusion criteria was patients aged over 18 at time of diagnosis and those diagnosed elsewhere.

**Outcome measures:**

Understanding clinical, biochemical, and histological AIH features in children, evaluating treatment responses, and reporting short- and long-term complications, including mortality.

**Results:**

Sixteen pediatric cases were diagnosed, with an average age of 9.84 ± 4.13 years. Females comprised 75% of patients, and 31.3% presented with acute liver failure. Jaundice was the most common symptom, and hepatosplenomegaly was observed in 18% of cases. Most patients had elevated transaminase levels, along with positive anti-smooth muscle antibody (ASMA) and antinuclear antibodies (ANA). Common hematological abnormalities included anemia (56.3%) and thrombocytopenia (37.5%). All patients underwent liver biopsy, with interface hepatitis present in 81.3% of cases. Treatment mainly involved prednisone and azathioprine. Three patients died, one discontinued therapy, two patients were lost to follow-up, and 10 remained on treatment.

**Conclusion:**

Autoimmune hepatitis affects Jordanian children, primarily female children. Jaundice is the most common presenting symptoms. Only Type I AIH occurred in our cohort. Although of good response to conventional treatment with steroids and immunosuppression, mortality reached 18.8%.

**Supplementary Information:**

The online version contains supplementary material available at 10.1186/s12887-024-04590-9.

## Strengths and limitations of this study

- Represents a comprehensive overview of AIH in Jordanian children.
- Provides a rare longitudinal analysis with long-term outcomes.
- Being a single-center study, it may not be universally applicable

## Introduction

Autoimmune hepatitis (AIH) is a progressive, inflammatory liver disorder of unknown etiology. If left untreated, the disease progresses to liver cirrhosis and liver failure [1]. The disease arises from a disruption in immune tolerance, leading to an autoimmune reaction that induces liver injury. This self-attack is triggered by T-helper cell-mediated liver autoantigen recognition and B-cell production of autoantibodies and persists due to impaired regulatory T cell number and function [2].

The diagnosis of AIH requires compatible histological findings and is supported by elevated serum aminotransaminase levels, elevated serum immunoglobulin G (IgG) levels, and/or positive serological marker(s), and ruling out viral, hereditary, metabolic, cholestatic, and drug-induced diseases that may resemble AIH [1]. According to serology, autoimmune hepatitis may be further divided into two subtypes: type 1, positive for antinuclear antibodies (ANA) and/or smooth muscle antibody (SMA), and type 2, positive for anti-liver-kidney microsomal antibody (anti-LKM-1) and/or anti-liver cytosolic antigen type 1 (anti-LC-1) [1].

Clinical manifestations of AIH vary in children and adolescents, and often presents acutely with a more aggressive course than in adults, unless treated promptly [3, 4]. Immunosuppression is the mainstay of AIH therapy. Prednisone is administered as initial therapy, either alone or in combination with azathioprine [4, 5]. Liver transplantation is indicated in patients who present with acute liver failure that is unresponsive to corticosteroids and in those patients who develop end-stage liver disease. Autoimmune hepatitis is an indication for liver transplantation in approximately 2%–3% of pediatric and 4%–6% of adult recipients in the United States and Europe. The treatment goal is to maintain aminotransferases at a normal level or less than twice the reference value and simultaneously reduce the inflammatory infiltrate within the liver.

At present, only sporadic reports on autoimmune hepatitis in children are available. Prior to this study, there has been a notable absence of research within Jordan that specifically examines autoimmune hepatitis in Jordanian children. Our study embarked on a comprehensive exploration of autoimmune hepatitis in children, delving into the clinical, biochemical, and histological facets, all while tracing the enduring impacts. All children diagnosed with this condition and closely monitored throughout the study period at King Abdullah University Hospital in Jordan were included.

## Methods

### Study design and participants

This was a retrospective review of pediatric patients diagnosed with autoimmune hepatitis according to the simplified diagnostic scoring system of the International Autoimmune Hepatitis Group [5] between 2015 and 2023 at King Abdullah University Hospital in Jordan. All patients diagnosed with autoimmune hepatitis and aged ≤ 18 years at the time of diagnosis were included. Patients older than 18 years at the time of diagnosis, those with no liver biopsies reviewed at our hospital, and those with missing data were excluded. The diagnosis of AIH was based on: compatible liver biopsy findings with supportive laboratory results (immunological markers and immunoglobulin levels, with exclusion of other infectious or metabolic differential diagnoses).

### Liver biopsy

Liver biopsies were performed by a qualified interventional radiologist. In case of bleeding tendency; correction of bleeding tendency using appropriate procoagulation products, after consultation with pediatric hematology team. INR of 1.7 considered acceptable for our interventional radiologist. Transjugular liver biopsies are not available in our hospital. None of the patients who underwent liver biopsy developed serious bleeding.

### Treatment regimen

In our unit, we follow the suggested Juvenile Autoimmune Hepatitis treatment protocol by the European Society for Pediatric Gastroenterology Hepatology and Nutrition (ESPGHAN) Hepatology Committee Position Statement [1].

Prednisolone at a dose of 2 mg/kg daily is the mainstay treatment in our unit. Steroid use is tapered over 8 weeks to 2.5–5.0 mg daily. Azathioprine is the standard steroid sparing agent. It is administered at a dose of 1–2 mg/kg to all patients after 2 weeks of steroid initiation. As thiopurine methyltransferase (TPMT) activity level and azathioprine metabolite levels are unable to be assessed in our unit, we depend on frequent blood counts to detect any toxicity. Mycophenolate (20 mg/kg twice daily) is our standard second line therapy. We require 3 years of biochemical and immunological remission and a liver biopsy with no inflammation to stop treatment.

### Data collection

The electronic medical records of patients were carefully reviewed to gather a wide range of information. This included details on their clinical presentation, physical examinations, and various laboratory data such as liver enzyme levels, total and direct bilirubin, complete blood counts, and markers related to liver function (Prothrombin Time, Albumin, Ammonia levels). Additionally, we collected data on immunological markers like ANA, antinuclear antibody. ASMA, anti-smooth muscle antibody. AMA, anti-mitochondrial antibody. anti-LKM-1, anti–liver-kidney microsomal antibody. anti-LC-1, anti-liver cytosolic antigen type 1infectious hepatitis markers (Hepatitis B surface antigen (HBsAg), Hepatitis C antibodies (anti HCV antibodies), Hepatitis B surface antibody (HBsAb), Hepatitis A virus Antibodies (IgM, IgG),(anti HAV IgM and IgG), Epstein-Barr virus (EBV) Antibodies (EBV Ab), cytomegalovirus (CMV) antibodies (CMV Ab), as well as findings from radiological tests such as Liver ultrasound with Doppler, Abdominal CT scan, and Magnetic resonance cholangiopancreatography (MRCP) if available.

We also compiled reports from liver biopsies, and organized all this information into prepared Excel data sheets encompassing every parameter. We documented any interventional therapies performed, which may have included upper endoscopies with or without variceal ligation, as well as the treatments administered, such as steroids, immunosuppressants, and supplements.

Our study also tracked patient outcomes, evaluating improvement through the normalization of liver enzymes, synthetic function, and immunological markers, and assessed the results of follow-up liver biopsies, if available. Remission was reported as biochemical, immunological and histological remissions. Additionally, we recorded the specific type of treatment received at the time of evaluation and any cases of mortality.

### Statistical analysis

Data were collected and entered into an Excel spreadsheet. Means and standard deviations (SD) of the data were calculated. The frequencies of the events and percentages were presented.

### Ethics

The study was approved by the Institutional Review Board and Research Committee of the Faculty of Medicine at the Jordan University of Science and Technology (20230126). As patient data were anonymous, the requirement for informed consent was waived.

### Patient and public involvement

Patients and/or the public were not involved in the design, or conduct, or reporting, or dissemination plans of this research.

## Results

Sixteen patients were included in this study. The median age at presentation was 9.8 ± 4.3 years (range 1.5–16 years). The majority of patients were female (12/16, 75%) (Table 1). All patients presented with the typical symptoms and/or signs (jaundice, emesis, abdominal distention, etc.) associated with AIH. The duration of symptoms ranged from 7 to 90 days, with an average duration of 31.3 days. The most common presenting symptoms included jaundice (75%), emesis (43.8%), and abdominal distention (43.8%). For a comprehensive overview of the prevalence of all symptoms, please refer to Table 1.

**Table 1:** Demographic and clinical features at presentation

Characteristics (*n* = 16)	Frequencies
Age at presentation (years)	
Average ± SD	9.84 ± 4.13
Age range	1.5 -16 years
Gender,	
Male	4 (25%)
Female	12 (75%)
Duration of symptoms prior to presentation (days)	31.3 days (7–90 days)
Clinical features at presentation:	
Jaundice	12 (75%)
Emesis	7 (43.8%)
Abdominal distention	7 (43.8%)
Edema	3 (18.8%)
Hepatomegaly	3 (18.8%)
Splenomegaly	3 (18.8%)
Excessive itching	3 (18.8%)
Bleeding	2 (12.5%)
Encephalopathy	2 (12.5%)
Acute liver failure	5 (31.3%)

The clinical and biochemical laboratory data of patients with AIH at baseline is shown in Table 2. Anemia was the most common finding observed in our cohort (9 patients, 56.3%), with an average hemoglobin level at presentation of 97 g/l (range 71–121 g/l). The causes of anemia were hypersplenism in three patients (33.3%) and bleeding/iron deficiency in three other patients (33.3%), while two patients (22.2%) had aplastic anemia and one patient (11.1%) had an immune-mediated anemia. Thrombocytopenia was observed in six (37.5%) patients in our cohort. The average platelet count was $179\times {10}^{9}/L$ $({\text{range}}16-348\times {10}^{9}/L$ ). Interestingly, eosinophilia (absolute eosinophilic count (AEC) > 500/mcL (> 0.5 × 10^9^/L)) was observed in four (25%) patients of our cohort. The AEC ranged between 512—9360/mcL.

**Table 2:** Baseline blood workup at presentation

Characteristics	Average (range)	Abnormal results N (%)
Hb (g/l)	97 (71–121)	9 (56.3%)
WBC (X 10^3^)	5.0(1.9–25.6)	5 (31.3%)
Platelet (× 10 ^6^)	179 (16–348)	6 (37.5%)
INR	2.05 (1.1–4.6)	9 (56.3%)
ALT (IU/L)	362( 16–1195)	15 (93.8%)
AST (IU/L)	465 (25–1675)	14 (87.5%)
Albumin (g/l)	34.1 (19–41)	9 (56.3%)
Bilirubin (mmol/L)	84 (4–288)	12 (75.0%)
Alkaline phosphatase (IU/L)	441.6 (151–1180^a^)	4 (25.0%)
GGT (IU/L)	92.7 (2–402)	9 (56.3%)
ESR (mm/hr)	58 (8–329)	6 (37.5%)

^a^The two patients with very high level of Alkaline phosphatase had concomitant vitamin D deficiency

Transaminase levels were almost always elevated; the average ALT level was 362 (16–1195 IU/L) and the average AST level was 465 (25–1675 IU/L). Alkaline phosphatase was elevated in four patients, and none of them were diagnosed with primary sclerosing cholangitis (PSC) or overlap syndrome. Although the erythrocyte sedimentation rate (ESR) was not available for all patients at presentation, it was elevated in 66.7% of patients, with an average rate of 65.2 mm/hr (Table 2).

Serology rates and histopathological features of patients are indicated in Table 3. According to the autoantibody profile, ANA, ASMA, and anti-mitochondrial antibodies (AMA) positivity were noted in 62.6%, 56.3%, and 30.8% of patients, respectively. Two patients had their antibodies tested (i.e., ANA, AMA, and ASMA), and all tests came back negative. Hypergammaglobulinemia was found in 9 out of 12 (75%) patients. Data regarding anti-LKM antibody assessment was available for only two patients and were negative.

**Table 3:** Serology rates and histopathological features of patients

Serology markers	Positivity rates
ANA	10 (62.5%)
AMA	4 out of 13 (30.8%)
ASMA	9 (56.3%)
Anti-LKM	0
Hypergammaglobulinemia	9 out of 12 (75.0%)
Histological features	
interface hepatitis	13 (81.3%)
Lymphocytic infiltrates	10 (62.5)
Lymphoplasmocytic portal infiltrates extending into lobule	7 (43.8%)
hepatocyte rosette formation	2 (12.5%)
- Degree of fibrosis (Ishak Method) ^a^ (0–6):	
- 0	- 5 (31.3%)
- 1	- 3 (18.8%)
- 2	- 1 (6.3%)
- 3	- 3 (18.8%)
- 4	- 2 (12.5%)
- 5/6	- 2 (12.5%)

^a^ Ishak K, Baptista A, Bianchi L, et al. Histological grading and staging of chronic hepatitis. *J Hepatol*. 1995;22(6):696–699

Viral serology (HAV IgM, HBsAg, HCV Ab, CMV Ab, EBV Ab) were universally negative in our cohort except for two patients. One patient in our cohort reported a positive HAV IgM six weeks prior to presentation, and one patient had positive CMV IgM 8 weeks prior to presentation.

All liver biopsies showed findings compatible with autoimmune hepatitis. Among the cohort, interface hepatitis was found in 81.3%, lymphocytic infiltrates in 62.5%, lymphoplasmacytic portal infiltrates in 43.8%, and hepatocyte rosette formation in 12.5% of patients. Initial liver biopsies showed variable degrees of fibrosis; while five patients showed no fibrosis, two patients had very advanced fibrosis at presentation (Table 3). A detailed description of liver biopsy findings are provided in a Supplementary file (Appendix 1).

The most commonly used radiological assessment was liver ultrasound with Doppler for the portal tract; most of the patients (12/16, 75.0%) had heterogeneous echotexture of the liver. MRCP was available for four patients; one of them showed findings consistent with cirrhosis, and the other three showed mild intrahepatic dilatation. None of them had findings suggesting PSC.

With regard to treatment options (Table 4), 15 (93.8%) patients were started on prednisolone, while azathioprine was added after two weeks (as per treatment protocol). Five patients experienced relapses after initial positive responses. Two of them experienced relapses while tapering steroids, so we returned them to their previous steroid dose and they did fine. The other three required a full steroid dose (2 mg/kg prednisolone) with tapering adjusted based on the liver enzymes follow-up results. Two patients switched to second line treatment (i.e., mycophenolate) (Table 4).

**Table 4:** Treatment offered for autoimmune hepatitis

Type of treatment	Frequency
Prednisolone	15 (93.8%)
Azathioprine	15 (93.8%)
Mycophenolate	2 (12.5%)
Other (Propranolol, Spironolactone, URSO^a^, Vitamin K,..)	6 (37.5%)

^a^One patient presented at first with persistent cholestasis post-hepatitis A infection. After further work-up; turned to be AIH. Her liver biopsy wasn`t suggestive of PSC or Overlap syndrome. UDCA stopped at that stage

**Fig. 1:**
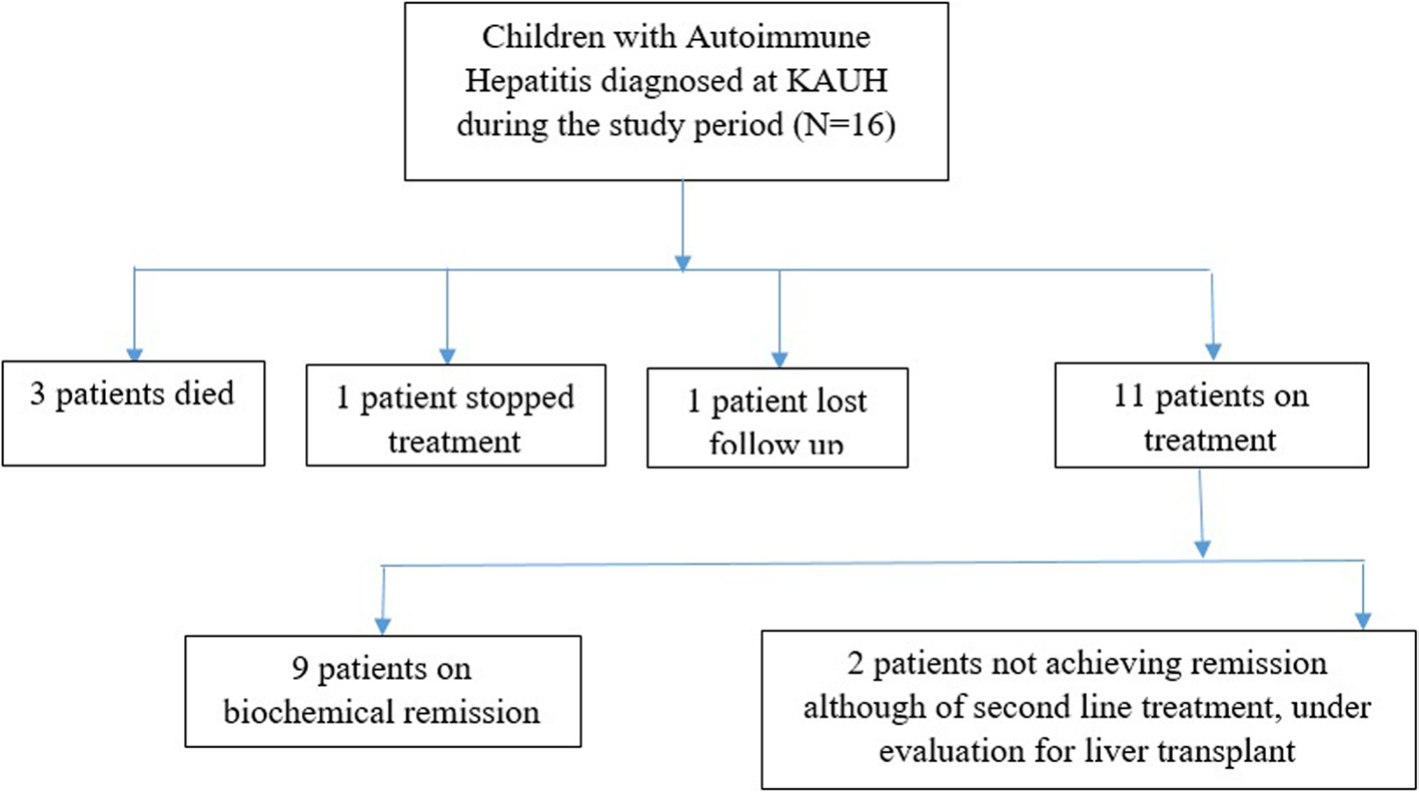
Summary of the patients’ outcome

Patient outcomes are listed in Table 5. The average follow-up period in our cohort was 114 weeks (range: 8–290 weeks). Eleven (68.8%) patients remained on treatment. Nine (81.2%) patients were in biochemical remission, while only three of them experienced immunological remission. One patient discontinued treatment and was doing well at the last follow-up, while another patient was lost to follow-up. Per the last follow-up, among the three patients who died, one patient presented with concomitant aplastic anemia and died due to a multisystem failure, and the other two died of liver cirrhosis (Table 5, Fig. 1).

**Table 5:** Patients` outcomes

Outcome	Frequencies
Development of Varices	6 (37.5%)
- Required ligation	- 3 out 6 (50%)
Treatment discontinued	1 (6.3%)
Biochemical remission (out of 10)^a^	9 (90.0%)
Immunological remission (out of 10)^b^	3 (30.0)
Histological remission (out of 4)	1 (25%)
Patients on treatment:	11 (68.8%)
- Treatment escalated	2 (18.2%)
- Treatment tapered	9 (81.2%)
Died	3 (18.8%)

^a^As per the last follow-up

^b^ Two of them have a biochemical remission also

## Discussion

In Jordan, autoimmune hepatitis is a liver disorder mainly affecting adults, with limited understanding of the disease in Jordanian children. This study aimed to fill this gap by analyzing clinical data of Jordanian children with AIH. Worldwide, AIH affects 100,000–200,000 people annually, with incidence rates of 0.9–2/100,000 and a prevalence of 11–25/100,000 [6]. In the US, the prevalence is estimated to be 31.2/100,000 [7]. Similar rates are seen in Denmark (23.9/100,000) [8] and Japan (23.4/100,000) [9], but the prevalence in Jordan is unknown. In Jordan University Hospital, 30 adult AIH cases were reported over six years [10]. Multicenter national studies are needed to estimate the true prevalence and incidence of AIH in our country.

AIH affects all ages but often presents more acutely in children and is mostly diagnosed before 18, peaking around 10 years old (mean age: 9.84 ± 4.13 years). This aligns with similar studies in Arabic children (7.2 ± 2.8 years in Egyptian children, 9.4 ± 4.2 years in Saudi Arabian children) [11, 12].

Patients with autoimmune hepatitis demonstrated a female predominance across all ethnicities. Several theories have been proposed to explain this phenomenon. Sex hormones influence innate immunity and activation of some genes on the X chromosome, in addition to epigenetic and microbiota factors, although the exact mechanism of such factors has not been fully elucidated [2, 13]. In our study, the disease was more common in females (75%), and this finding is consistent with that of other studies [1, 2, 6, 11, 12, 14–16].

Patients with AIH have variable presentations ranging from insidious to acute and

fulminant. Autoimmune hepatitis can be asymptomatic in 25% of patients [15]. Symptoms and signs can differ in severity among AIH patients, varying from asymptomatic to fulminant hepatic failure. Moreover, AIH presents with an acute onset (duration < 30 days) in 25%–75% of patients [17]. In our study, the average symptom duration was 31.3 days (range 7–90 days). Children with AIH often present with non-specific symptoms (viral-prodrome) such as anorexia, nausea, abdominal pain, and malaise [17]. The most common symptom was jaundice (75%) in our study, consistent with findings in studies conducted in Iran [16], Egypt [11] and Pakistan [18]. Notably, none of the patients in our study were asymptomatic.

The prevalence of cirrhosis at initial diagnosis varies globally. In Pakistan [18] and India [19], clinical cirrhosis was observed in 68% and 71% of patients, respectively. A US center reported a 55% cirrhosis rate in 2010 [20]. Within our cohort, 18.8% presented with cirrhosis, akin to a Danish cohort (28.3%). Sample size limitations might influence these rates, rather than differences in access to care or differences among ethnicities.

AIH diagnosis involves transaminase elevation, detectable autoantibodies (ANA or SMA), and elevated serum IgG levels. Type 1 AIH is associated with ANA/SMA, while type 2 involves the LKM-1 antibody. In White North American adults, 80% had ANA, 63% SMA, and 3% anti-LKM-1 at presentation, with 51% having multiple autoantibodies. Detecting two autoantibodies improved diagnostic accuracy from 58 to 74% [21]. Pediatric studies show varied antibody positivity (ANA: 30%–100%) [22, 23]. Arab countries reported ANA (around 68%) and SMA (70–84%) positivity [11, 12], which aligns with our findings (ANA: 62.6%, SMA: 56.3%).

In AIH, marked IgG elevation is common, distinguishing it from other liver diseases. Approximately 85% of patients with AIH have elevated IgG levels, dropping to 60–75% in acute-onset cases. Elevated IgG levels have a high sensitivity (90–98%) and specificity (approximately 96%) for AIH diagnosis [24, 25]. Our study indicated elevated IgG levels in 75% of patients at presentation.

Elevated serum transaminases (AST, ALT) and gamma globulin are common markers in autoimmune hepatitis, but alkaline phosphatase elevation is less frequent. While transaminase levels may not indicate disease severity, they are useful for monitoring disease activity. Biochemical remission is often used to assess treatment response [26]. Severe cases may demonstrate high bilirubin levels [17]. In this study, transaminases were consistently elevated.

Thrombocytopenia in autoimmune hepatitis is rare, and its cause remains unclear [27]. A study in Egypt showed that 23.5% had anemia, 29.4% had thrombocytopenia, and 8.8% had pancytopenia [11]. Interestingly, anemia was common in our cohort, and nearly 40% had thrombocytopenia, even without notable portal hypertension.

Typical AIH displays distinct features on biopsy: interface hepatitis, portal inflammation, plasma cells, hepatocyte rosette, and emperipolesis. Interface hepatitis involves an intense inflammatory infiltrate crossing the liver's boundaries. A liver biopsy is strongly recommended for diagnosis confirmation, ruling out other causes, and assessing fibrosis extent [28].

Emperipolesis, witnessed in 65% of AIH patients, correlates with severe necroinflammation and advanced fibrosis [29]. In this study, liver biopsies from all patients consistently revealed findings indicative of autoimmune hepatitis.

Prednisone, often with azathioprine, is the primary and effective treatment for AIH [15]. Alternative medications like budesonide, mycophenolate mofetil, cyclosporine, tacrolimus, and ursodeoxycholic acid are considered for steroid non-responding cases or those with steroid contraindications [30].

Remission in AIH is defined as normalized liver tests and IgG levels, symptom disappearance, and histological signs, typically lagging behind biochemical remission by months [14]. Early azathioprine use in children helps mitigate corticosteroid-related side effects. Drug withdrawal is considered after prolonged remission, but relapse rates range from 60 to 80% [31].

Regular, lifelong follow-ups are essential post-treatment, even after remission maintenance. Studies in Egypt, Saudi Arabia, and India have reported varying remission rates and relapse tendencies [32–34]. In this study, 15 patients received prednisolone with azathioprine; two switched to mycophenolate due to azathioprine resistance. Only one patient stopped treatment with no relapse. Biochemical remission was achieved in 9 out of 11 patients in our cohort. As no liver transplant service is available in Jordan, none of our patients received a liver transplant, although two patients were evaluated for a transplant abroad.

This study is the first to explore autoimmune hepatitis in Jordanian children, offering insights into clinical findings, serological markers, treatments, and treatment outcomes within this cohort. However, this study has some limitations. While the study was conducted in a significant referral center in North Jordan, the findings of this single-center study may not fully represent Jordan. Additionally, the rarity of AIH and the small number of cases could restrict the generalizability of the findings. Furthermore, limited data availability, particularly for anti-LKM, was a notable limitation, with only two patients having this data available.

## Conclusion

Our study sheds light on autoimmune hepatitis in Jordanian children, revealing its occurrence and essential characteristics in this specific population. We also noted a higher prevalence among female patients, consistent with international trends. Clinical presentations and treatment responses resembled findings from diverse geographical regions, highlighting global consistency in AIH characteristics.

However, our study, as a single-center retrospective approach, has limitations including a relatively small sample size and limited follow-up duration. Acknowledging these constraints, there is a crucial need for future prospective, multicenter studies. The mortality rate within our cohort and the presence of patients awaiting liver transplant signal a critical need to establish a dedicated liver transplant program. This imperative move would signify a national investment aimed at mitigating suffering and curbing the substantial costs associated with seeking such interventions abroad.

### Supplementary Information

**Additional file 1.**

## Data Availability

No datasets were generated or analysed during the current study.

## Abbreviations

AIH: Autoimmune hepatitis
ANA: Antinuclear antibody
ASMA: Anti-smooth muscle antibody
AMA: Anti-mitochondrial antibody
Anti-LKM-1: Anti–liver-kidney microsomal antibody
Anti-LC-1: Anti-liver cytosolic antigen type 1
PSC: Primary sclerosing cholangitis
HBsAg: Hepatitis B surface antigen
MRCP: Magnetic resonance cholangiopancreatography
