# Supplementary material for: Retrospective, single-center analysis of autoimmune hepatitis in Jordanian children: clinical features, treatments, and outcomes
Source: BMC Pediatr. 2024 Feb 8;24:102. doi: 10.1186/s12887-024-04590-9 (PMC10851525; doi:10.1186/s12887-024-04590-9)
Supplement: Supplementary file 1 — Additional file 1. [file 12887_2024_4590_MOESM1_ESM.docx]

Appendix 1: Detailed histological features of liver biopsies in our cohort:

| Patients | Interface Hepatitis | Lymphocytic  Infiltrate | Lymphoplasmocytic portal infiltrates extending into lobule | Hepatocyte rosette formation | Emperipolesis | Chronic Hepatitis | Degree of Fibrosis (out of 6) |
| --- | --- | --- | --- | --- | --- | --- | --- |
| 1 | • |  | • |  |  |  | 0 |
| 2 | • | • |  |  |  |  | 3 |
| 3 |  |  | • |  |  |  | 0 |
| 4 | • |  |  |  |  |  | 0 |
| 5* |  |  |  |  |  | • | 0 |
| 6 | • | • |  | • |  |  | 0 |
| 7 | • |  | • |  |  |  | 5 |
| 8 | • | • |  |  |  |  | 4 |
| 9 | • | • | • |  |  |  | 3 |
| 10 | • |  | • |  |  |  | 5 |
| 11 | • | • | • |  |  |  | 4 |
| 12 | • | • |  |  |  |  | 3 |
| 13 | • | • |  |  |  |  | 1 |
| 14 | • | • |  |  |  |  | 1 |
| 15 | • | • | • |  |  |  | 1 |
| 16 |  | • | • | • | • |  | 2 |

*Known case of Systemic Lupus Erythematosus (SLE), liver biopsy was done while patient on immunosuppressants.
